# Supplementary material for: Genome analysis of biosurfactant producing bacterium, Bacillus tequilensis
Source: PLoS One. 2023 Jun 2;18(6):e0285994. doi: 10.1371/journal.pone.0285994 (PMC10237388; doi:10.1371/journal.pone.0285994)
Supplement: Attachment — Submitted filename: Response to Reviewers.docx [file pone.0285994.s009.docx]

## RESPONSE LETTER

**Ref.: Whole Genome Sequencing and Annotation of Biosurfactant Producing Bacillus tequilensis**

**Dear Editors,**

We appreciated the review of our manuscript and the suggestions given to make it suitable to **PLOS One** journal which is in high quality standards. As requested, this letter responds to each point raised by the reviewers. Please, kindly note that changes were marked up in the manuscript and are transcribed herein:

Editor Comments

1. Title should be changed as "Genome analysis of biosurfactant producing bacterium, Bacillus tequilensis.

**Response:** We appreciate the careful evaluation of our manuscript and the recommendations made to raise the quality of it. As advised by the editor, we would like to let the editor know that the title of the manuscript has been changed to "Genome study of biosurfactant generating bacteria, *Bacillus tequilensis*." Thanks.

2. All microorganisms name should be italics.

**Response:** We value the in-depth analysis of our manuscript and the recommendations made for enhancing its quality. We would want to let the editor know that all of the microbe names have been changed to italics as per the editor's suggestion. Thanks.

3. Data related to genome-based taxonomy including average nucleotide identity and digital DNA-DNA hybridization with closely related type strains should be provided.

**Response:** We value the thorough analysis of our manuscript and the suggestions offered to improve its quality. We would like to inform the editor that in section 2.1 first paragraph, we have added the genome-based taxonomy including ANI (Average Nucleotide Identity) analysis and digital DNA-DNA hybridization/ Genome – Genome comparison by GGDC(Genome Genome Distance Calculator) with closely related type strains of *Bacillus tequilensis* as suggested by the editor. Thanks.

4. These lines are incorrect "we concluded that Bacillus tequilensis produces biosurfactants and the novel isolate was deposited in GenBank with accession number KU529483". You have submitted nucleotide sequence of 16S rRNA gene to the Genbank not bacteria. Similar mistakes are in whole manuscript.

**Response:** We value the thorough analysis of our manuscript and the suggestions offered to improve its quality. We would want to inform the editor that we have changed this throughout the manuscript as suggested by the editor. I appreciate you pointing out this error.

5. Manuscript is too lengthy; it should be concise. It should be focused only on biosurfactant related genes.

**Response :** We appreciate the in-depth evaluation of our manuscript and the recommendations made to raise its quality. Since the work involves a whole genome analysis and complete annotation of a bacteria, we would want to let the editor know that we made every effort to keep the paper as concise as possible. As a result, the text became a little lengthy. We made an effort to discard a few information, such SSR identification. Let us know if there are any other analysis need to be deleted. Looking forward to hear for the suggesion.

6. Experimental data related to biosurfactant production in this strain are necessary.

**Response :** We value the thorough analysis of our manuscript and the suggestions offered to improve its quality. We would like to inform the editor that, we have carried out a number of experimental studies including the Haemolysis test, oil spreading test, CTAB agar plate test, Drop collapse test etc from which we have concluded that the Bacillus tequilensis isolate used in this investigation produces biosurfactants. This study has already been published, so we've used it as a source in result session 2.2 in the present manuscript.The present study is a continuation of the earlier investigation.The publication can be access from the folowign link.

[**https://link.springer.com/article/10.1007/s13762-018-2089-9**](https://link.springer.com/article/10.1007/s13762-018-2089-9)
